# Ecological Risk, Input Flux, and Source of Heavy Metals in the Agricultural Plain of Hebei Province, China

**DOI:** 10.3390/ijerph19042288

**Published:** 2022-02-17

**Authors:** Kui Cai, Chang Li

**Affiliations:** 1 Institute of Geological Survey, Hebei GEO University, Shijiazhuang 050031, China; kcai@hgu.edu.cn; 2 Hebei Key Laboratory of Strategic Critical Mineral Resources, Hebei GEO University, Shijiazhuang 050031, China; 3 School of Economics and Management, Hebei University of Science & Technology, Shijiazhuang 050018, China

**Keywords:** heavy metals, pollution assessment, input flux, source, management

## Abstract

A large amount of heavy metal (HM) inputs exists in the farming areas of the Hebei plain of northern China. However, the potential ecological risk, source, and input flux of HMs in these areas have not been well-investigated. In this study, atmospheric deposition, fertilizer, irrigation water, and agricultural soil samples were collected from farming areas (~74,111 km^2^) in Hebei Province, China. The HM index of geoaccumulation (I_geo_) and potential ecological risk index (RI) of soil was calculated for eight HMs. The source and input flux of each element were predicted using the input flux and principal component score–multiple linear regression (PCS–MLR) methods. The results showed that Cd and Hg increased I_geo_ values, and the maximum levels of As (29.5 mg/kg), Cu (228.9 mg/kg), Cd (4.52 mg/kg), and Zn (879.0 mg/kg) were greater than the health risk screening values in the soil quality standard of China. The potential ecological risk factor (Er) of Cd demonstrated a moderately potential ecological risk, accounting for 67.72%. The distribution map showed that Cd was mainly concentrated in eastern area of Baoding (BD) in the study area. The result of the atmospheric dry and wet deposition contributed more to soil pollution than the usage of fertilizer or irrigation water by calculating the input flux. The order was Zn (94%) > Cu (92%) > Pb (89%) > Cr (86%) > Cd (72%) > Hg = Ni (71%) > As (59%). Principal component analysis (PCA) results showed that there were four sources of HMs in soil. Geological sources contribute to the accumulation of As, Cr, and Ni in soil. Cu and Pb in the soil were attributable to the input from vehicular emissions and irrigation water. Cd and Zn in the soil were attributable to the farming activity, whereas Hg originates from the combustion of coal. The results of PCS–MLR demonstrated that the contribution rate of As, Ni, and Cr in the study area was 30.06%, 71.86%, 57.71% for the first group (natural source); Cu, Pb and Zn were 71.78%, 63.59%, and 30.72% for the second group (vehicle emissions); Zn was 60.93% for the third group (fertilizer application and irrigation water); and Hg was 85.16%, for the fourth group (coal combustion). These factors provide a valuable reference for remediating HM pollution.

## 1. Introduction

In agroecological environments, heavy metal pollution (HMP) is attributed to improper application of chemical fertilizers and pesticides, as well as irrigation water and industrial factors [1,2,3]. The dispersion of HMP over large areas has become a hotspot in environmental management. China is primarily a country having a large agricultural production; therefore, there are concerns about environmental problems related to agriculture [4,5]. Agricultural pollution influences food safety, which affects human health. HMP in agricultural soils, in addition to the quality and safety of agricultural products, has recently become an essential topic that should be considered to protect farmlands. In a soil pollution survey, the standards were breached at 19.4% of sites; i.e., 13.7%, 2.8%, 1.8%, and 1.1% of the considered area was slightly, mildly, moderately, and considerably polluted, respectively. Trace elements, such as Cd, Ni, Cu, As, Hg, and Pb, were the major pollutants [6]. Therefore, the overall situation was not good. The quality of arable land and soil is worrisome; moreover, pollution levels are high in soils near abandoned industrial sites and mines. China issued novel soil environmental quality standards and guidelines to mitigate risks on agricultural land (GB15618-2018) [7]. Based on the standards, measures must be taken to appropriately manage the soil environment on agricultural lands [8].

Industry and agriculture form the economic foundation of Hebei Province. However, industrial activity developments cause serious HMP, thereby limiting the agriculture economy. For example, Cd (0.92 mg/kg) and Hg (0.0725 mg/kg) were major toxic elements in the smelting of a middle area in the Hebei plain, China [9]. The heavy metal (HM) concentrations of Cd, Hg, Pb, Zn, Cu, Cr, and Ni were 1.86, 0.29, 154.78, 496.17, 91.06, 131.7, and 40.99 mg/kg, which exceeded the background values in street dust around an industrial zone in Shijiazhuang (SJZ) [10].

Based on a multiobjective geochemical survey conducted to identify pollutants from industrial, mining, and agricultural activities, the fertilizer usage rate, livestock, poultry manure, pesticides, irrigation water, atmospheric deposition of industrial waste and automobile exhaust, and other human activities were the primary causes of soil pollution [11,12].

The usage of chemical fertilizers has attracted considerable attention because fertilizers are major contributors to inorganic agricultural pollution. A 2018 statistical yearbook demonstrated that ~3.3 million tons of chemical fertilizers were used in Hebei Province from 2011 to 2017. Fertilizers are used inefficiently because their usage rate is only 30–40% [12]. Unused fertilizers are dispersed in soil and groundwater. The atmospheric deposition of pollutants is another concern. Atmospheric dry and wet deposition accounts for 43–85% of the total As, Cr, Hg, Ni, and Pb inputs. Note that >50% of Cd, Cu, and Zn inputs are attributable to livestock and poultry manures [13]. Moreover, a major water shortage can be observed in the Hebei plain, China (the study area); therefore, there is a major contradiction between supply and demand in agricultural production. The surface water is polluted; hence, groundwater is the primary source of irrigation water for agricultural production. Thus, agricultural pollution is a serious problem in the Hebei plain. Consequently, it is essential to establish annual inputs of HMs in the agricultural soil in Hebei Province for controlling and reducing HMP.

The primary pollutants of agricultural nonpoint sources (chemical fertilizer usage, livestock and poultry farming, and rural solid waste) can be understood using geographic information system [14], SWAT [15], AGNPS [16], export coefficient [17], input flux [18], and other HMP research models. To examine the soil pollution sources, the principal component score–multiple linear regression (PCS–MLR) method [19,20] and positive matrix factorization (PMF) method [21,22,23] are used for analyzing the contribution rate of HMs to soil pollutants. In this study, soil samples and three types of medium samples, including samples subjected to fertilizer, irrigation water, and atmospheric deposition, were obtained from the Hebei plain. An input flux method was used to determine the primary input fluxes associated with HMs for atmospheric deposition, fertilizer, and irrigation in agricultural soils. Moreover, the source apportionment (PCS–MLR) was used to analyze the contribution level of each pollution source with respect to the HMs. Hence, the aim of this study is to (1) analyze HMs concentration, the HMP level, and potential ecological risks of Hebei Plain, China, caused by industry and agriculture, (2) evaluate HMs spatial distribution characteristics, (3) calculate the HMs input fluxes of fertilizers, irrigation water, and atmospheric depositions to agricultural soils, (4) identify the pollution source and contribution rate of HMs observed in the study area via principal component score–multiple linear regression (PCS–MLR), and (5) provide valuable management to control the pollution source. We will provide valuable data for controlling and remediation HMP with respect to Hebei agricultural soils.

## 2. Materials and Methods

### 2.1. Study Area

**Figure 1:**
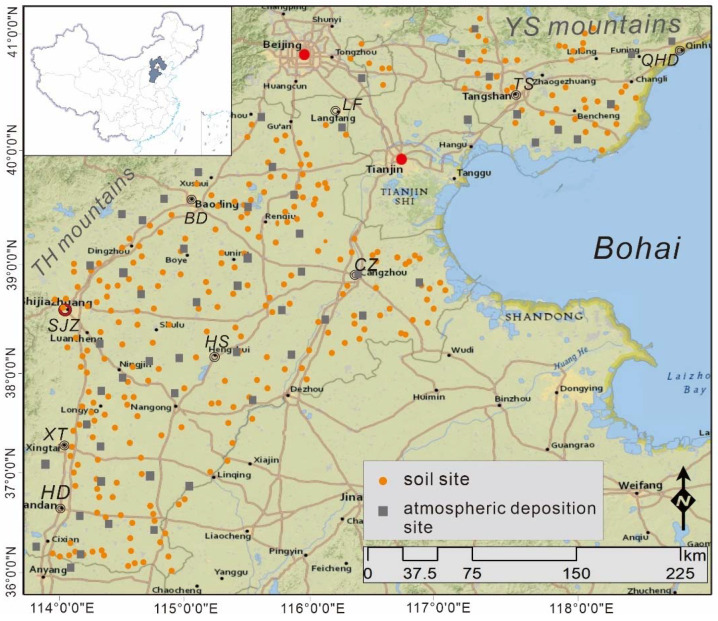
The sampling site of soil and atmospheric deposition in Hebei plain, China.

The Hebei plain covers an area of ~7.4 × 10^4^ km^2^ and encompasses both Jidong (JD) and Jizhongnan (JZN) plains (Figure 1). The Qinhuangdao (QHD) and Tangshan (TS) cities are located on the JD plain. The Langfang (LF), BD, Cangzhou (CZ), SJZ, Hengshui (HS), Handan (HD), and Xingtai (XT) cities are located on the JZN plain. The Hebei plain is bound by the Yellow River to the south, Yanshan (YS) Mountains to the north, Taihang (TH) Mountains to the west, and the Bohai Sea to the east. The Haihe and Luanhe River systems are located on the Hebei plain. The terrain varies in elevation from ~100 m in the west to ~3 m along the Bohai Coast. The region has a temperate monsoon climate, and climate changes are evident with a warm summer and a cold and dry winter. The regions between the foothills and coast contain moist and brown soils having clear variations. Moist soil can be observed in the middle of the plain as well as along the YS Mountains to the north and TH Mountains to the west. The farmland on the east side of the plain contains both Eutric Cambisols and Eutric Luvisols, whereas saline–alkali soil can be observed in the coastal region. The farmland covers an area of 6.5 × 10^4^ km^2^, of which 4.46 × 10^4^ km^2^ is irrigated. The grain output in 2019 was 3.41 × 10^7^ tons, which primarily comprised wheat, rice, and maize [12]. The JD plain’s main industry is steel and cement. The JZN plain’s main industry is steel, coal, cement, and metallurgy. Meanwhile, the sewage irrigation is more serious.

### 2.2. Sample Collection and Treatment

#### 2.2.1. Soil Samples

A total of 287 soil samples were collected from June to September in 2012 from the top 20 cm of the arable layer in the farming areas. The surface soils, weeds, roots, gravels, bricks, fertilizer clumps, and other debris were removed during sampling. To ensure representativeness of samples, one point was considered to remain fixed during sampling. Furthermore, three to five subsamples were obtained at multiple points within a range of 20 m to obtain a sample with a weight of >1 kg. After natural air drying, gravels, biological debris, and plant roots were extracted. Subsequently, the samples were passed via a 20-mesh nylon screen, mixed, and ground to −200 mesh. Note that ~100 g of the samples was packed and sent for analysis [24].

#### 2.2.2. Atmospheric Deposition

In this study, 60 sampling barrels were arranged in the farmland areas of the entire study area, including 12 samples in the JD plain and 3 counties in the north of LF and 48 samples in the south of the JZN plain. They were carried to the sampling sites after being treated using distilled water. When arranging the sampling sites, they were placed on a rural roof, ~5–8 m above the ground. Anhydrous ethanol was placed in a barrel at a 1-cm depth to prevent the deposition from producing secondary dust and avoid industrial areas and highways. The tank was placed in a room to allow natural evaporation, and then, the dry sediment was extracted. After weighing and recording, the samples were sealed, numbered, and sent to the laboratory for testing.

#### 2.2.3. Chemical Fertilizer

Based on field surveys, well-known local chemical fertilizers were determined and used as samples. A total of 19 types of compound fertilizers, 17 types of urea, and 5 phosphate fertilizers were collected. Then, the application amounts of various types of chemical fertilizers were recorded, and the samples were sealed, numbered, and directly sent to the laboratory for analysis. A total of 41 samples were collected.

#### 2.2.4. Irrigation Water

Two hundred and thirty-two samples were stored in a polythene plastic pot (the inner plug must be plastic) having a milky white rectangular stopper and a volume of 1 L. The polyethylene pot containing water samples was soaked in 10% HNO_3_ for three days before being filled with groundwater samples. Subsequently, the pot was cleaned with tap water and distilled water. The sample bottles were washed three to five times using water samples before sampling. Then, the samples were sent to the laboratory for analysis.

The analysis and measurements were performed in strict accordance with standard GB/T 5750.6 [25]. For more details of the analysis and measurement methods, please refer to previous studies [24,26].

### 2.3. Chemical Analysis and Quality

We weighed a 0.25-g soil sample and placed it in a Teflon beaker, added 5 mL of HCL, heated it at low temperature on an electric heating plate, added 2 of mL HNO_3_, HF, and HClO_4_, continued heating and cooling, transferred it to a 25 mL colorimetric tube, diluted it with water to a scale, and stirred well. For more details of the process, please refer to DZ/T0279-2016 [26]. Cr, Cu, Ni, Pb, and Zn in the supernatant were analyzed via inductively coupled plasma atomic emission spectrometry (X Series II, Thermo Electron Corporation, Waltham, MA, USA). Cd was analyzed using a graphite furnace atomic absorption spectrometer (PerkinElmer PinAAcle 900T, Perkin Elmer Instruments (Shanghai) Co., LTD, Shanghai, China), and As and Hg in the supernatant were measured using a hydride generation atomic fluorescence spectrometer (AFS-2202E, Beijing HaiGuang Instrument Co., Ltd., Beijing, China). Moreover, for determining the pH value using an ion-selective electrode method, refer to DZ/T 0279.34-2016; for determining cation exchange capacity using a Hexamminecobalt trichloride solution/spectrophotometric method, refer to HJ 889-2017); and for determining organic carbon (orgC) using a potassium dichromate oxidation–external heating method, please refer to LY/T 1237-1999.

The precision and accuracy of experimental tests were evaluated using standard samples, recovery tests, indoor and outdoor repeat samples, and coded samples. The accuracy of deposition and solution samples can be controlled as per the national standard of substances GBW [26]. The average relative error ((Relative error/Absolute error) × 100%) of all samples was <4%. The precision of sample analysis was less than that specified in the standard; the precision rate was 100%. The precision and accuracy of all samples agreed with the requirement for Regional Ecogeochemical Evaluation [25].

### 2.4. Statistical Analysis

The HMs were subjected to descriptive statistical analysis using SPSS. In the analysis, the minimum, maximum, mean, correlation analysis, and input flux were determined in the study area. All data must be transformed via logarithmic transformations if a normal distribution is not obtained before Pearson’s correlation analysis. The spatial distribution of the HMs was obtained using ARCGIS version 10.5. Principal component analysis (PCA) results were obtained via factor analysis (FA) using SPSS. Subsequently, the PCS–MLR results were obtained. The extraction of As, Cu, Pb, Cd, Ni, Cr, Hg, and Zn was 0.726, 0.846, 0.797, 0.978, 0.943, 0.898, 0.97, and 0.983, respectively.

### 2.5. Pollution Assessment Methodology

#### 2.5.1. Index of Geoaccumulation

The index of geoaccumulation (*I*_geo_) was calculated for each metal to assess soil pollution [27]. *I*_geo_ allows the impact of human activity on the soil environment to be distinguished from natural factors [28] and is essential for identifying the pollution source. *I*_geo_ can be calculated as follows:

(1) $$I_{geo}=log_{2}\left[\frac{C_{n}}{1.5\times B_{n}}\right]$$

where *C*_n_ is the concentration of metal *n* in the farm soil (mg·kg^−1^) and *B*_n_ is the background value of metal *n* (mg·kg^−1^). A correction factor of 1.5 was used to consider natural fluctuations in the background value because of lithographic variations. The *B*_n_ values for Hg, Cd, As, Cu, Ni, Pb, Cr, and Zn were 0.04, 0.11, 12.8, 21.8, 30.8, 21.5, 68.3, and 71.9 mg·kg^−1^ [29], respectively. The contamination level based on *I*_geo_ belonged to one of the following classes: *I*_geo_ ≤ 0 (clean), 0 < *I*_geo_ ≤ 1 (slight), 1 < *I*_geo_ ≤ 2 (mild), 2 < *I*_geo_ ≤ 3 (moderate), 3 < *I*_geo_ ≤ 4 (moderate-heavy), 4 < *I*_geo_ ≤ 5 (heavy), and *I*_geo_ > 5 (severe).

#### 2.5.2. Potential Ecological Risk Index (RI)

Lars Håkanson—a Swedish scientist—proposed the potential ecological risk index RI [30], which quantitatively shows the response observed for basic elemental abundance and the synergistic effect of pollutants. Currently, it is primarily applied to assess the HMP in soil and RI. This factor considers the potential ecological risk of a single HM and the integrated ecological effect of various HMs [31]. Thus, the corresponding risk level could be obtained. Equation (2) was then used to calculate the RI for each metal.

(2) $$\mathrm{RI}=\sum_{i}^{m}E_{r}^{i},\;\mathrm{where}\;C_{f}^{i}\;=C_{i}/C_{n}^{i}\;\mathrm{and}\;E_{r}^{i}\;=T_{r}^{i}\times C_{f}^{i}$$

where $C_{f}^{i}$ is the pollution coefficient of HM *i*, *C_i_* is the concentration of HM *i* (mg·kg^−1^), $C_{n}^{i}$ is the preindustrial reference value of the substance for HM *i* [30], and $T_{r}^{i}$ is the toxicity coefficient of HM *i*. $T_{r}^{i}$ values were 40, 30, 10, 5, 5, 5, 2, and 1 for Hg, Cd, As, Cu, Ni, Pb, Cr, and Zn, respectively. $E_{r}^{i}$ is the potential ecological risk factor Er for HM *i*, which belongs to one of the five following categories: <40 (slight); 40–80 (moderate), 80–160 (strong), 160–320 (very strong), and >320 (extremely strong). RI shows the comprehensive index for all HMs. RI was classified into five: <150 (slight), 150–300 (moderate), 300–600 (strong), 600–1200 (very strong) and >1200 (extremely strong).

### 2.6. Input Flux Analysis

#### 2.6.1. Atmospheric Deposition

Sixty sampling bottles were arranged in the study area. The average representative area of each bottle was 1.35 × 10^9^ m^2^. The amounts of HMs in the agricultural land due to atmospheric deposition can be calculated as follows:

(3) $$Q_{a,i}=\frac{C_{i}\times W\times K}{S}$$

where *Q_a,i_* is the amount of HMs (*i* = As, Cd, Cr, Cu, Ni, Pb, Zn, and Hg) present in the study area because of atmospheric deposition (mg·m^−2^·y^−1^), *Ci* is the content of HM *i* because of atmospheric deposition (mg·kg^−^^1^), *W* is the annual amount of deposition obtained based on the sampling bottle (kg), *K* is the conversion coefficient (10,000 m^2^), and *S* is the area in which the individual soil sample can be observed (706.5 cm^2^).

#### 2.6.2. Irrigation Water

The amounts of HMs in agricultural land that can be attributed to the irrigation water were estimated based on the volume of irrigation water used annually and the heavy metal concentrations in water as per Equation (4).

(4) $$Q_{w,i}=C_{i}\times V\times 10^{-4},$$

where *Q_w,i_* is the annual input of the HM *i* from irrigation water (mg·m^−2^·y^−1^), *C*_i_ is the concentration of the HM *i* in the irrigation water (g·L^−1^), and *V* is the volume of irrigation water used (m^3^·ha^−1^·y^−1^).

#### 2.6.3. Fertilizer

The content could not be easily calculated by homogenizing various fertilizers because of differences between various fertilizers and their complex formulations. Thus, we calculated the input flux of each fertilizer based on the amount of fertilizer applied per year and the sum using Equation (5).

(5) $$Q_{f,i}=\overset{n}{\underset{\begin{array}{c} i=1\\ j=1 \end{array}}{\sum }}M_{i,j}\times C_{i,j}\times 10^{-4},$$

where *Q_f,i_* is the total HM input obtained from fertilizer *j* (mg·m^−2^·y^−1^), *M_i,j_* is the total amount of fertilizer *j* applied (kg·ha^−1^·y^−1^), and *C_i,j_* is the concentration of HM *i* in fertilizer *j* (mg·kg^−1^).

The total input fluxes of HM *i* (*Q_t,i_*) from atmospheric deposition, irrigation water, and fertilizer were calculated as follows [32]:

(6) $$Q_{t,i}=Q_{a,i}+Q_{w,i}+Q_{f,i}$$

### 2.7. Source Apportionment Methodology

PMF [21,22] and PCS–MLR [19,20] are examples of source apportionment methods [33]. Recently, the latter has been applied to soil. Because of the complexity of soil systems, the contributions of different sources to the overall concentration of a given element cannot be easily estimated quantitatively. PCS–MLR has enormous application for soil source; it may be applicable in local areas with similar geological conditions (such as diagenetic processes, parent materials, soil types, and landforms). Recently, researchers successfully tracked the origin of HMs in soil via PCS–MLR [19,20,34]. The fundamental assumption associated with PCS–MLR is that the total element concentration was linearly correlated with the contribution of different sources. The basic principle has been described by Thurston and Spengler (1985) in detail [33].

Therefore, in this study, PCS–MLR was used to assess the contribution of HMs from different sources to explain the variation in the HM concentration of the agricultural soil. The current PCS–MLR method has been modified based on Thurston and Spengler (1985) [33] (generally, considerable differences can be observed with respect to the concentrations of different elements). Therefore, data are converted in a dimensionless standard form as follows:

(7) $$Z_{i,j}=\frac{C_{i,j}-\mu_{i}}{\sigma_{i}}$$

where *i* = 1, 2, …, n and *j* = 1, 2, …, m are the total numbers of HMs and samples, respectively; *Z_i,j_* is the standardized value of element *i* for sample *j*; *C_i,j_* is the concentration of element *i* for sample *j*; *μ_i_* is the mean concentration of element *i*; and *σ_i_* is the standard deviation of the concentration distribution of element *i* [34].

First, the normal distribution of variables was tested. The results showed that all variables were normally distributed before PCA. The PCA with a varimax rotation transform is applied to normalized data, and the PCA is calculated for the rotation. These PCA are related to multiple sources influencing the local soil position. The initial-rotated PCA of each component was used to estimate the absolute PCS (APCS) as per the method proposed by Thurston and Spengler (1985) [33] to obtain an improved proportional relation with the corresponding source contribution.

The regression of the normalized elemental data of the APCS provides a coefficient that can be used to convert APCS into the source contribution to the sample. The equation is as follows:

(8) $$Y_{j}=X_{0}+\overset{a}{\underset{i=1}{\sum }}X_{i}APCS_{i,j}$$

where *Y_j_* is the standardized value of the concentration of the HM *j*; *APCS_i,j_* is the rotated absolute component score for component *i* of element *j*; *X_i_* APCS*_i,j_* is the contribution of element *j* by the source identified with component *i*; and *X*_0_ is the contribution by sources not considered in PCA. An *X*_0_ approximately equal to 0 shows successful PCA–APCS [34]. Based on regression results, coefficients were employed to calculate the contribution of a pollution source with respect to the HMs in the study area.

## 3. Results and Discussion

### 3.1. Soil HM Concentration and Pollution Indices

Table 1 shows descriptive statistical data for pH, orgC, CEC, and HM concentrations in soil in the study area. The mean values of pH, orgC, CEC were 8.15, 1.03%, and 11.23 cmol/kg. The mean values of Cu, Pb, Cd, Hg, and Zn were higher than the local background values. In particular, Cd was twice the background value. The ranges of As, Cu, Pb, Cd, Ni, Cr, Hg, and Zn were 2.47–29.50, 5.60–228.90, 13.7–125.70, 0.05–4.52, 5.40–43.20, 25.00–112.10, 0.01–0.36, and 15.80–879.00 mg/kg, respectively. The maximum values of As, Cu, Cd, and Zn were greater than soil risk screening values for the Chinese Standard [35], indicating that certain samples were polluted in the study area. Especially for Cd, the maximum value was 90.4 times the minimum value. Moreover, the coefficient of variation of Cd was 183.02%. The main reason was that two samples with highest content (4.16 mg/kg and 4.52 mg/kg) originated from the eastern part of BD and led to a high coefficient of variation. The result of Cd concentration is similar with Zhou (2021) who reported that the maximum value of Cd in arable soil of eastern area of BD was 3.83 mg/kg, 96.67% of the samples that have exceeded the soil risk screening values [36].

Table 2 lists the *I*_geo_ and RI mean values. When *I*_geo_ > 0, the HMs in the soil primarily originate from human activities rather than the natural source. When the *I*_geo_ results showed that the mean values of the HMs were <0, no pollution owing to human activities can be observed in all samples. However, the *I*_geo_ mean values of Cd and Hg were very close to 0. The sample numbers of Cd and Hg were considerably greater than zero and accounted for 43.86% and 32.63% of the total, respectively, indicating that the majority of Cd and Hg originate from human activities. However, the *I*_geo_ values were >0 for Pb, Zn, and Cu at 9, 6, and 13 samples, respectively, indicating that the human effects must be seriously considered, although they were smaller than those observed for Cd and Hg. The mean *I*_geo_ values of As, Cr, and Ni were 99.30%, 98.95%, and 100% lower than zero, indicating the natural origin of these elements. The result is similar to the result previously reported in Cai (2020). Moreover, Cai (2020) also showed that As was controlled by Fe and Mn oxide [23]. The mean *I*_geo_ values of HMs in the soil of the study area were as follows: Cd (−0.008) > Hg (−0.05) > Pb (−0.14) > Cu (−0.15) > Zn (−0.18) > Cr (−0.19) > Ni (−0.24) > As (−0.34).

The Er and RI results indicated that Hg has the highest Er value, thus resulting in a (more) moderately potential ecological risk compared with that associated with the remaining HMs in the study area. This Er value accounts for 99.65% of that associated with the total sample. For Cd, the Er value showed a moderately potential ecological risk, accounting for 67.72% of that associated with the total sample. Meanwhile, the corresponding value of Er of the two samples (4.16 mg/kg and 4.52 mg/kg) was 1134 and 1233. It has reached extremely strong risk levels. The remaining HMs, such as As, Cr, Ni, Pb, and Zn, did not show potential ecological risk values except for one sample in which Cu achieved a moderate ecological risk. However, the comprehensive RI risk analysis demonstrated that 82.46% of the samples reached moderate risk levels. The risk levels were observed to be extremely strong, very strong, and strong in two, three, and seventeen samples, respectively.

### 3.2. Soil HM Spatial Distribution

#### 3.2.1. As

**Figure 2:**
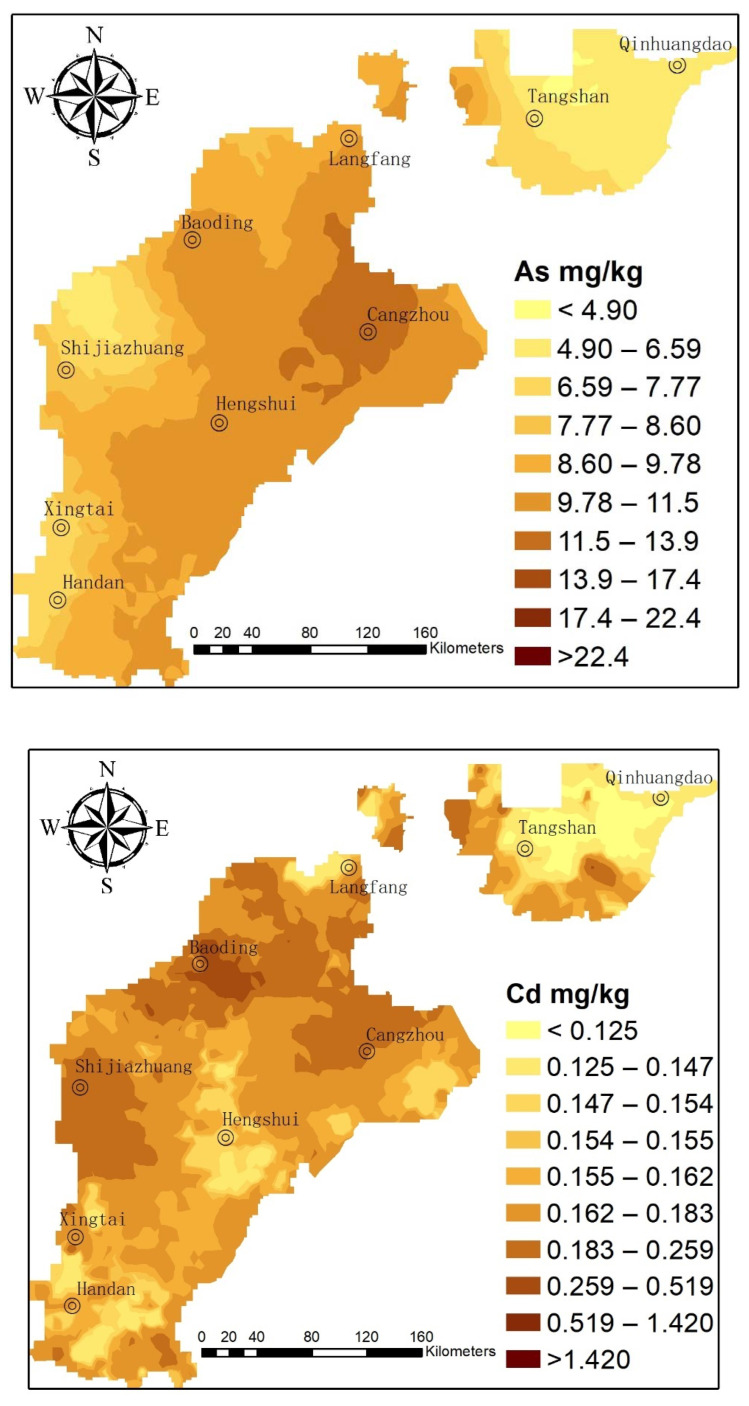

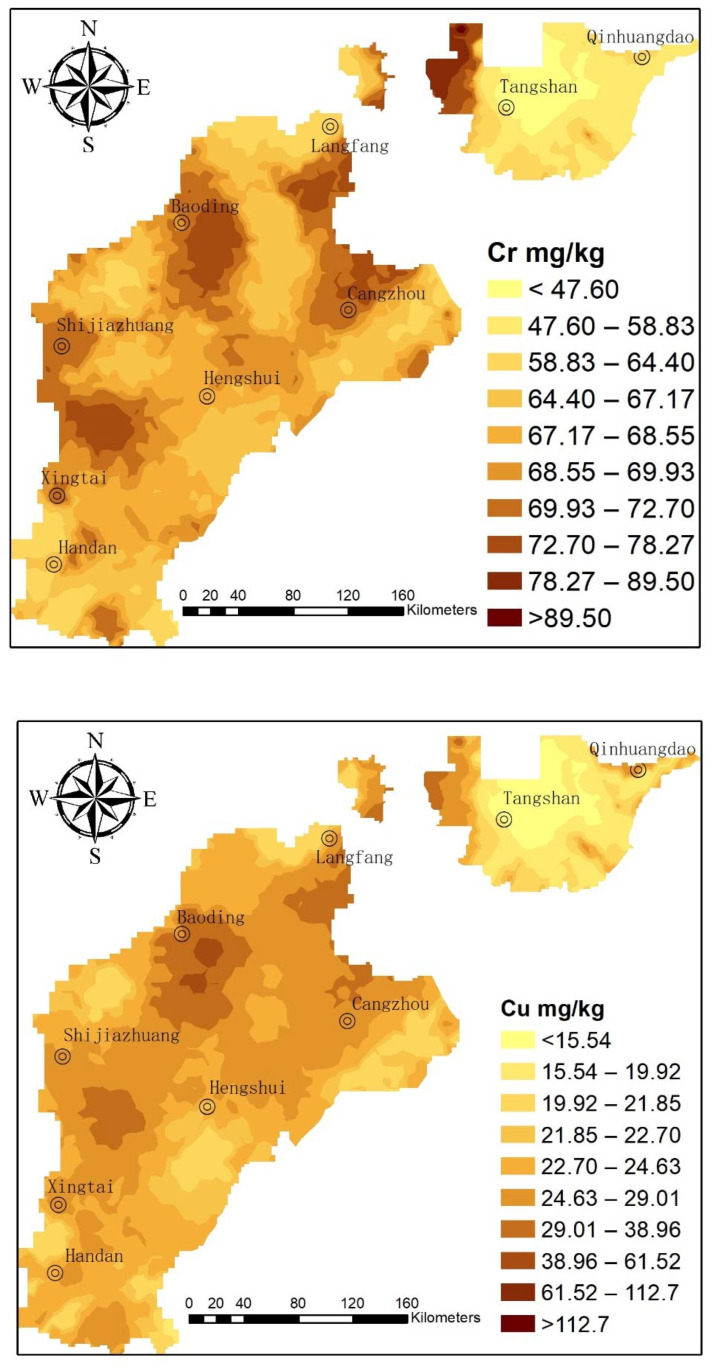

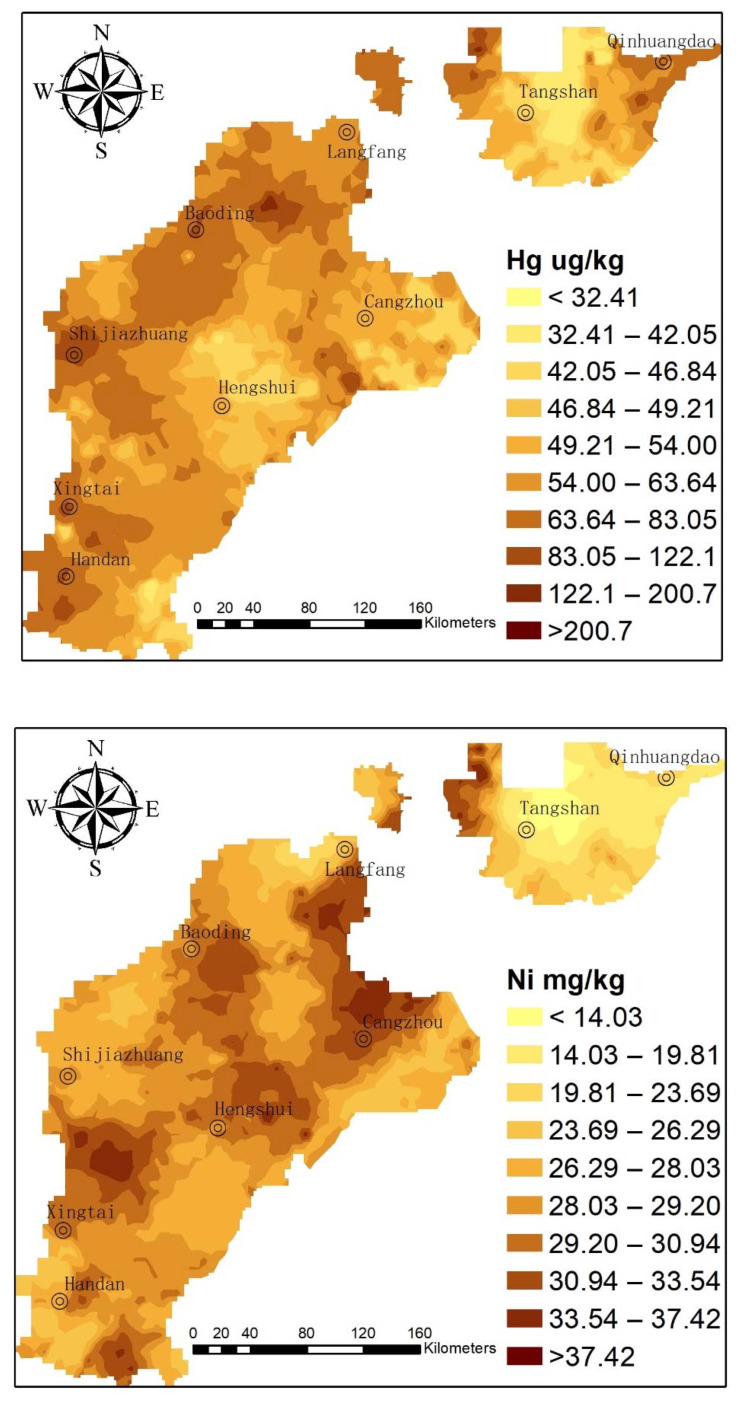

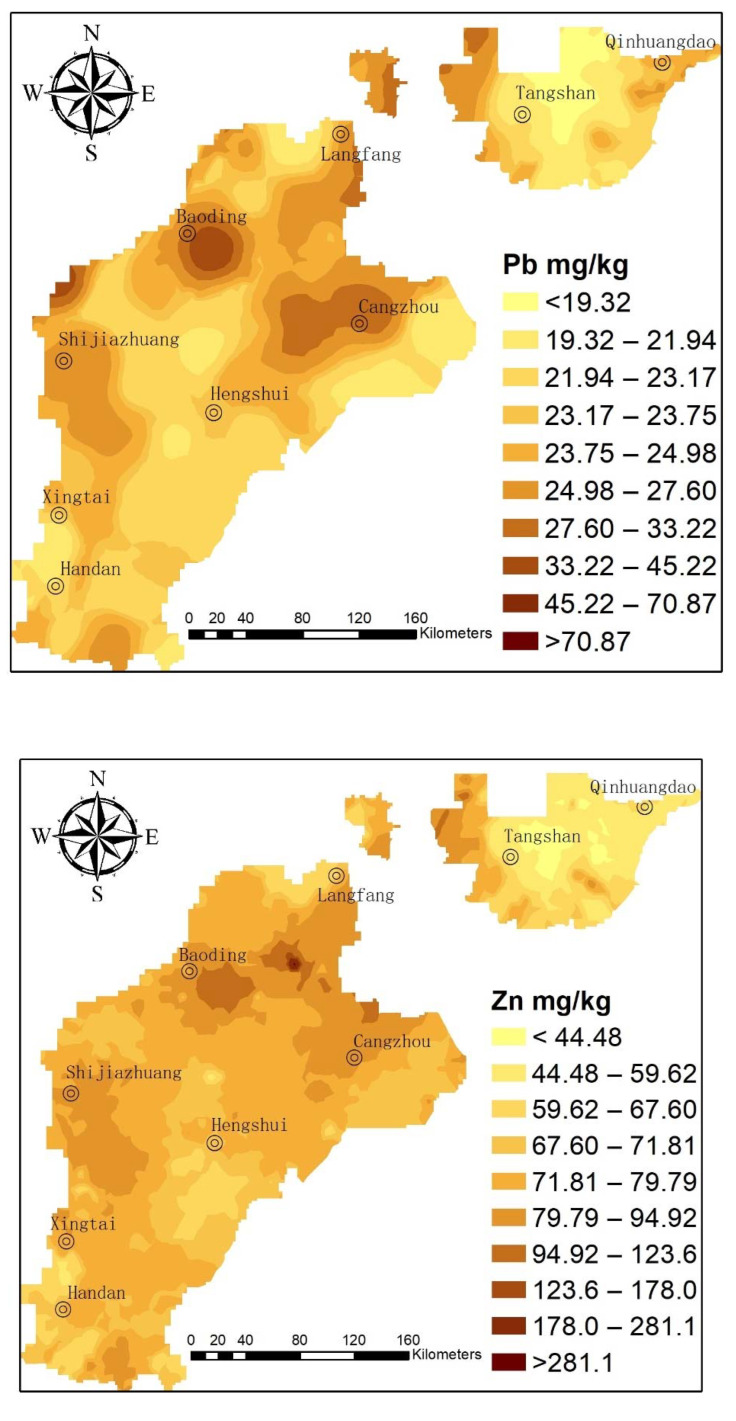
Prediction map of soil HMs in the Hebei plain prepared via an ordinary Kriging method.

Figure 2 shows the distribution map of As in the study area. The distribution trend demonstrated that the mountain front has lower As than the middle-plain and seaside areas. Higher soil As concentrations were reported in the northeast part of the study area. The high-value areas were primarily distributed in the west and north part of CZ with (pH > 8.5) soil and the eastern parts of BD. A large number of scholars indicated that pH was one of the key factors affecting As [23,37]. For example, Shen (2020) reported that the content of bioavailable forms of As increased gradually with the increase of pH [37].

In the eastern part of BD, the accumulation of As due to metallurgy and sewage irrigation has affected food security [9,38,39]. Wang (2021) reported that the abnormal high values of As were mainly caused by the sewage irrigation, the mean value of As has risen to 23.69 mg/kg [9]. Zhou (2021) showed that As was also affected by industrial emissions in addition to the weathering process of geological parent rock in the BD aera [36].

#### 3.2.2. Ni

From Figure 2, the surface soil of the study area mostly contained low Ni content (Table 1), indicating that the spatial distribution of Ni in the piedmont of the TH Mountains was better than that of Ni in the YS Mountains. The distribution pattern of Ni in the northeast of the central plain was lower than that in the other areas. The low-value environment was primarily distributed in TS and certain areas toward the east of the study area. Ni was mainly affected by natural geological background factors in the Hebei plain [9,38].

#### 3.2.3. Cr

According to Figure 2, the Cr content in the soil from the study area was low (Table 1). The Cr distribution in the front of the YS Mountains was lower than that in front of the TH Mountains. High-value zones were observed in BD, south of CZ, south of SJZ, and LF. The average value of Cr was close to the background value, primarily indicating a natural origin. Moreover, Cr and Ni had very similar distribution trends. The result shows that Cr and Ni have the same source. Wang (2021) and Guo (2021) examined HMP in BD and Xiong’an New District of Hebei plain and demonstrated that Ni and Cr varied in a small range, thus representing natural soil formation background [9,38].

#### 3.2.4. Pb

As shown in Figure 2, the Pb content in most surface soil of the study area was relatively low. The high-value area was primarily distributed in BD, whereas the low-value area was located in TS and QHD. Many industrial and mining enterprises involved with the smelting, manufacturing, and usage of Pb products are located around BD and surrounding areas. Hence, the high Pb content in this area is attributable to the Pb-containing wastewater, waste gas, and waste residue discharged by such enterprises [39]. During gasoline combustion, Pb enters the atmosphere via the exhaust gas discharged by an automobile and then enters the surface soil through atmospheric settlement. Therefore, a high-value area of Pb can be observed in the jurisdiction of BD [40,41].

#### 3.2.5. Cu

As shown in Figure 2, the Cu concentration in the surface soil of the study area was slightly higher than that in most areas. The Cu concentration in the surface soil of the YS Mountains was lower than that of the TH Mountains. The overall distribution pattern showed a low trend in the NNE belt to the north of HD. A high-value area could be observed in the BD area of Hebei Province. Metal processing, machinery manufacturing, steel production, and other such enterprises were distributed around the city in the high-value zone [9,40]. The waste residue, waste gas, and wastewater discharged by these enterprises were the primary reasons for the presence of Cu in the surface soil.

#### 3.2.6. Hg

As shown in Figure 2, areas having high Hg content in the surface soil were primarily distributed in the surrounding regions of SJZ, BD, XT, and HD in Hebei Province. The mountain front showed higher Hg values than the coast. A higher value could be observed in front of the TH Mountains compared with that of the YS Mountains. Low-value areas were mostly distributed in the east area of TS. In the northern cities, coal-fired gas was used for heating during the winter. The deposition of Zn-containing compounds in dust and soot during combustion was the primary cause of Hg accumulation in the soils of the abovementioned areas [42,43].

#### 3.2.7. Cd

The Cd content in southern Hebei was higher than that in northern Hebei (TS–QHD). Cd-rich zones were located in the east area of BD, and the surrounding regions. These areas may have been affected because of smelting and practice of wastewater irrigation that has been followed for decades [41]. The Cd content in the area was higher than the background value of the soil. Cui (2014) reported the wastewater irrigation as the main factor influencing higher Cd concentration in BD soils [39]. Zhou (2021) and Guo (2021) also have indicated that smelting was the main reason contributing to Cd and other HM concentration in BD soils [36,38].

#### 3.2.8. Zn

Figure 2 shows that the Zn content in the surface soil of the study area was high in the south, low in the north, high in front of the TH Mountains, and low in the front of the YS Mountains, SJZ, Anyang, BD, and CZ. Industrial enterprises involved in various activities, including smelting processing, machinery manufacturing, galvanizing, instrumentation, organic synthesis, and paper-making, were located in the abovementioned high-value zone. The Zn accumulation in local soils is attributable to the metal smelting and waste emissions of these enterprises. Zhou (2021) considered that smelting contributed to Zn concentration in BD soils [36]. Guo (2021) reported similar results in the same area [38].

In summary, the Hebei province has been producing coal, iron and steel, metallurgy, and sewage irrigation for 40 years as a large industrial province [40]. Irrational development of industry and agriculture has resulted in the distribution characteristics of HMs in agricultural soil. The contents of As, Cr and Ni in agricultural soil were basically consistent with the background values of soil. However, due to the difference in pH value, leading to the high-value region of As, it was more inclined to the region with higher pH. The high-value distribution of Hg was concentrated in areas with concentrated coal-burning activities. The distribution of high values of Cd, Cu, Pb and Zn were mainly concentrated in the eastern and southern parts of BD due to wastewater irrigation and smelting.

### 3.3. Input Flux

**Figure 3:**
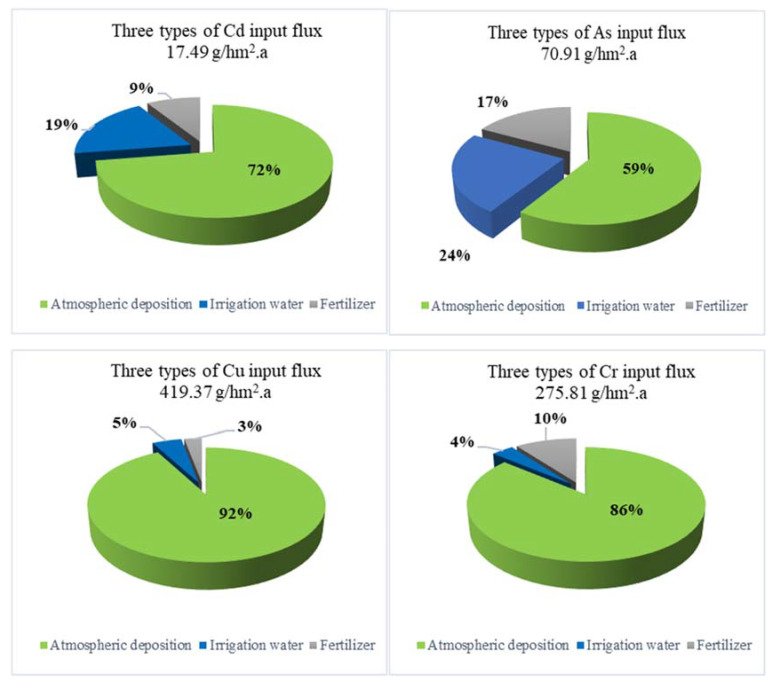

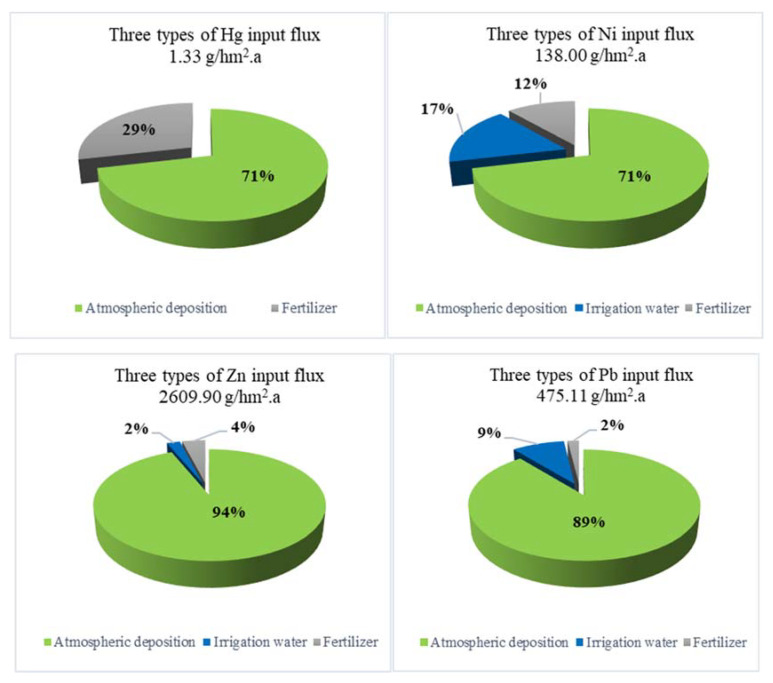
Contributions of three input types to agricultural land in the Hebei plain, China.

Figure 3 shows the results of the input fluxes of HMs in the Hebei plain. The input fluxes contributions of HMs of atmospheric deposition in the Hebei plain were very significant. The order was Zn (94%) > Cu (92%)> Pb (89%)> Cr (86%) > Cd (72%)> Hg (71%) = Ni (71%) > As (59%). The contribution rate of 24% and 29% of irrigation water and fertilizer were higher for As and Hg. Hou (2014) reported that irrigation water contributing 60–71% of the total inputs was the main source of metals (As, Cd, Cu and Hg), atmospheric deposition account for 72% and 84% of the total inputs was an important source of Zn and Pb, in the Yangtze River delta, China [18]. Hence, the contribution rate of As and Hg is smaller than that in the Yangtze River delta, China. However, the contribution rate of Zn and Pb is higher than that in the Yangtze River delta, China. In addition, the input fluxes of As, Cd, Cr, Cu, Pb, Zn in the study area are much higher compared with the mean value of As 28.0 g/hm^2^·a, Cd 4.0 g/hm^2^·a, Cr 61.0 g/hm^2^·a, Cu 108.0 g/hm^2^·a, Hg 1.4 g/hm^2^·a, Pb 202.0 g/hm^2^·a, Zn 647.0 g/hm^2^·a of China, except Hg 1.33 g/hm^2^·a [44].

Moreover, Figure 2 shows that HMs have regional and industrial characteristics. In particular, the chemical industry base close to CZ results in large input fluxes of As, Cr, Cu, Hg, Ni, and Pb. The input fluxes of Cd were the largest in the north LF, and the largest input flux of Zn could be observed in the north of TS where mining and metallurgy industries could be observed.

A large high-input area can be observed with respect to Ni, Pb, and other elements. In addition to CZ, as can be observed in the urban area of LF, Hg can be observed in the east of SJZ, and Pb can be observed in the urban areas of HS and Wuyi county, thus forming a high-input area because of the high contribution rate of atmospheric deposition.

The atmospheric deposition resulted in a high Cd input zone toward the north of LF and a high Zn input zone in the south and north of TS. This was because the Cd input obtained via local atmospheric deposition reached 130 g/hm^2^·a, which was ten times the average input flux (12.84 g/hm^2^·a) in the remaining study areas. Meanwhile, the value was much higher than the mean value of Cd 4.0 g/hm^2^·a of China [44]. The proportion of input flux of atmospheric deposition was 72%. Therefore, the atmospheric deposition had a major influence on the Cd distribution in the study area.

In all, the fertilizer input fluxes percentage for HMs ranged from 3% to 29%. The input flux percentages of irrigation water for HMs ranged from 2% to 24%, except for Hg. The input flux of Hg majorly comes from atmospheric deposition and fertilizer. Input flux percentages for different HMs also varied greatly (Figure 3). The input flux of most elements provided via irrigation water and fertilizer was smaller than the atmospheric deposition flux obtained via irrigation water and fertilizer, indicating that the groundwater quality in the Hebei plain was good. Furthermore, the high-value sites of the HM with respect to the input fluxes of irrigation water were scattered in the central and southern parts of the study area. The regions with high input fluxes of HMs in chemical fertilizers were observed in areas having developed agricultural production. Thus, the input fluxes of chemical fertilizers in the piedmont plain of the TH Mountains were considerably higher than that in CZ, TS, and other coastal areas. In the area, the use of fertilizers has led to HMs entering agricultural soils, and had a significant influence on the quality and safety of arable land. Compared to the results of Hou (2014) and Jiang (2014), the contribution rates and input fluxes for all HMs in the arable soil were significantly different in the study area. The main reasons might be: (1) other input pathways should be considered, such as agrochemicals, livestock manures, and sewage sludge. (2) The types and dosages of fertilizers were different in the three study areas. (3) The majority of farmland is wheat field in the study area, less irrigation water is applied in the agricultural production than in the other two study areas.

### 3.4. Correlation Coefficient

We evaluated the spatial distribution map of HMs as per the distribution trend of each element. Table S1 shows Pearson’s correlation coefficients for HMs for the soil samples obtained from the agricultural soil of Hebei plain. All samples were observed, i.e., Zn–Cd > Cr–Ni > Cu–Pb > Ni–As > Cd–Cu with a value of >0.60 (Table S1). The result of Pearson’s correlation coefficients indicated that they may have a similar source of Zn and Cd, Cr and Ni, Cu and Pb, Ni and As, Cd and Cu.

### 3.5. Contribution Rate

#### 3.5.1. FA

FA was employed to assess the As, Cu, Pb, Cd, Ni, Cr, Hg, and Zn source identifications with respect to the 287 soil samples obtained from the agricultural soil of Hebei plain.

**Figure 4:**
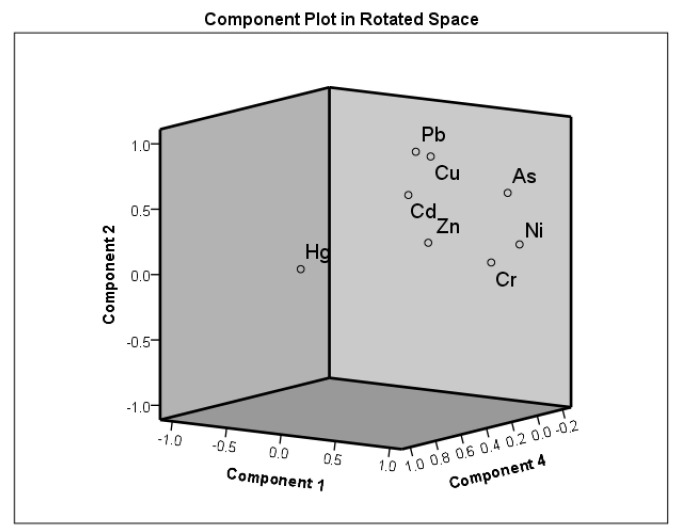
Each component plot in the rotated space map of HMs in the study area.

PCA was used as the extraction method. Varimax rotation was performed, and Kaiser normalization was employed. The Kaiser–Meyer–Olkin test value was ~0.60, indicating that FA was reasonable. Moreover, Bartlett’s sphericity test result, which was <0.01, confirmed the suitability of data for FA. Table S2 shows the rotated component matrix. Four primary factors with eigenvalues of >1 could be observed, accounting for 89.26% of the total variance (Figure 4). The first factor primarily involves As, Ni, and Cr, indicating that they originate from natural sources because the mean value approached the background value. The second factor involves Cu and Pb, the third factor is dominated by Cd and Zn, and the fourth factor is Hg. All target HMs for PCA were obtained from natural and anthropogenic sources. Cu and Pb associated with the second factor were primarily released to the environment from the exhaust and nonexhaust traffic-related emissions, including fuel combustion, fuel additives, erosion of the asphalt material, tire attrition, smelting, and brake wear. Zhou (2021) and Guo (2021) demonstrated that the Cu and Pb pollution is caused by metal smelting, metal waste and debris processing, and battery manufacturing [9,43]. Cd and Zn, which dominate the third factor, were derived from other human activities [45,46,47]. The considerable variations of Cd and Zn concentrations in soil samples confirm the usage of anthropogenic resources, including phosphatic fertilizers. The fourth component, i.e., Hg, primarily originated from the burning of coal, as previously indicated by Zhou et al. (2011) [48] and Huang et al. (2018) [41]. Moreover, Hg is the primary contributor to coal combustion in China.

#### 3.5.2. Source Distribution

**Figure 5:**
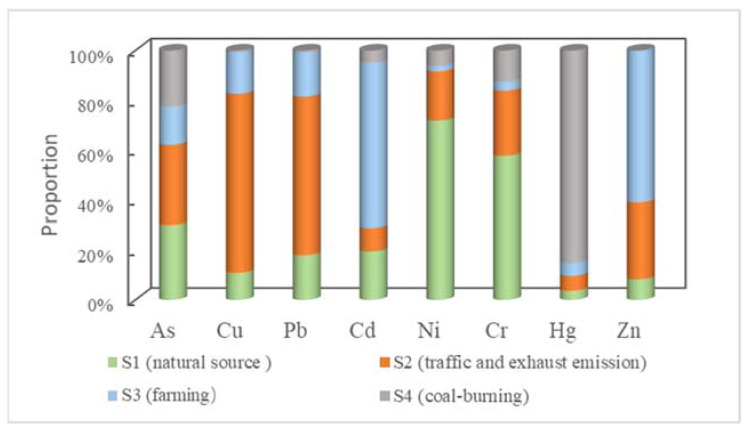
Contribution of HMs from four pollution sources estimated using PCS–MLR: S1 (natural source), S2 (vehicle emissions), S3 (fertilizer application and irrigation water), and S4 (coal combustion).

As per the sources and contributions of the abovementioned pollutants, agricultural soil pollution shows obvious multisource characteristics (Figure 5). Table S3 summarizes the concentration of HMs in fertilizers, irrigation water, and atmospheric depositions. Phosphate fertilizers contribute the most to the Zn and Cd amount, followed by compound fertilizers. Urea was the lowest contributor; hence, the usage of phosphate fertilizers was considered to be another component. The amounts of HMs, such as Pb, Zn, Cu, Pb, Cd, and Hg, obtained via atmospheric deposition were considerably higher than other elements in the soil, except for As, Ni, and Cr. The agricultural atmospheric deposition of HMs is primarily attributable to industrial waste and coal burning. Therefore, S3 and S4 are defined as farming and coal-burning groups, respectively.

The PCS–MLR results demonstrated that the contribution rate of As, Cu, Pb, Cd, Ni, Cr, Hg, and Zn in the study area was 30.06%, 10.09%, 17.94%, 19.48%, 71.86%, 57.71%, 3.73%, and 8.29%, respectively, for the first group; 32.01%, 71.78%, 63.59%, 9.33%, 19.76%, 26.15%, 5.99%, and 30.72%, respectively, for the second group; 15.38%, 16.62%, 17.63%, 66.32%, 2.30%, 3.66%, 5.12%, and 60.93%, respectively, for the third group; and 22.46%, 0.69%, 0.84%, 4.87%, 6.09%, 12.48%, 85.16%, and 0.06%, respectively, for the fourth group.

The contribution of As to the first and second group exceeded 30%, which was attributed to the natural source based on the correlation between the mean concentration level (close to the background) and Cr and Ni. The contribution of As to the third group was >15%, indicating multiple pollutions caused by the contribution of As in the soil. The contributions of Cu and Pb to the second group were >60%, indicating the major role of transportation and sewage water. The contributions of Cd and Zn to the third group were >60%, indicating the high contribution of fertilizers and industrial waste emissions. The contributions of As and Pb were >15%, whereas the contribution of Hg to the fourth group was >85%, indicating the high contribution of the coal combustion. The contribution of As was >20%, indicating that coal combustion releases a considerable amount of As. The results are similar to those of Zeng (2001) and Tian (2009), who reported that As originates from coal combustion [49,50]. The results indicated that all investigated HMs in the agricultural soil of Hebei plain were contributed by S2, S3, and S4 pollution sources. Therefore, a study should be conducted to examine how their release can be reduced by controlling the sources.

### 3.6. Management

As per the abovementioned research results, the agricultural HMP in the study area has become a serious problem, affecting the sustainable development of agriculture. Agricultural HMP usually refers to the large-area pollution caused by the unreasonable application of chemical fertilizers and pesticides, irrigation water, industrial and agricultural wastes, and household garbage during the agricultural production process. Therefore, the local environment department must take corresponding measures to solve this issue.

#### 3.6.1. Fertilizer

According to statistics, 3.124 million tons of fertilizers were used in 2008, which increased to 3.356 million tons in 2014 and then decreased to 3.22 million tons in 2017 [12]. The application of chemical fertilizers has decreased, although the output of wheat and corn has increased, indicating that the usage of chemical fertilizers has increased. However, the current efficiency of domestic fertilizers was significantly different from that of foreign fertilizers. Therefore, the local area should scientifically and reasonably control the usage of chemical fertilizers and pesticides, particularly phosphate fertilizers [51,52]. We suggest focusing on new high-efficiency fertilizers and developing high-efficiency and low-toxicity products, such as pesticides, from agricultural waste. Furthermore, we must achieve green prevention as well as develop a control technology and an inexpensive treatment technology for livestock and poultry manure and agricultural straw [53,54,55]. The content standards of various harmful elements in chemical fertilizers should then be formulated and improved.

#### 3.6.2. Coal Combustion

The coal usage decreased by 244.18, 316.96, and 281.05 million tons in 2008, 2013, and 2016, respectively. The coal usage rate has decreased via control in recent years. Industrial waste emissions have increased from 3755.8 billion cubic meters in 2008 to 7857 billion cubic meters in 2016 [12], i.e., almost doubling in eight years. Continuously increasing waste emissions will inevitably cause significant harm to the atmosphere and agricultural soil. Note that >70% of the coal was used for coal-fired power generation. The global Hg hazards associated with coal combustion and pollution caused by tens of millions of tons of coal gangue piles, spontaneous combustion, and rainwater leaching have considerably affected the society and environment. The evaluation of hazards associated with typical high-Hg coal was an urgent requirement with respect to environmental protection. The Hg content of coal in the study area was 0.14 mg/kg and that observed when burning coal was 0.15–0.16 mg/kg in various industries and processes such as power plants and coking [56]. In certain processes, the Hg content was considerably high. The environmental hazards caused by coal utilization cannot be ignored. Currently, the control technology of coal-fired mercury emissions is in the experimental development stage globally, and its commercial technology has not yet been completely developed [56,57]. Therefore, the importance to achieving inexpensive coal-fired Hg emission control is to develop a technique and provide technical and theoretical support for China’s policy-making departments to propose reasonable and effective control measures for coal-fired Hg. The internal laws of coal-fired Hg, harmfulness of emissions, and mineral composition and morphological characteristics of high-Hg coal combustion should be explored [58,59,60]. Therefore, the selection of typical high-Hg coal for combustion emission test research and analysis of its precipitation rules are important for achieving environmental protection in China and the rational development and usage of coal resources.

#### 3.6.3. Irrigation Water

The proportion of irrigation-water input was less from the perspective of the irrigation-water input flux. However, in certain regions, the level of HMs in sewage water irrigation should be considered. For example, in SJZ and BD, the Cd level in sewage irrigation was high [61,62]. The low surface concentration and high concentration of As were considerably related to groundwater [20]. Therefore, the level of HMs at both places should be considered before wastewater irrigation, and the water quality must meet the water quality standards for irrigation. In areas where wastewater irrigation has been applied for a long time, the content and accumulation rate of HMs in the soil, groundwater quality, residents’ physical health, and surrounding ecological environment should be monitored.

El-Mageed (2021) reported that adding Si could reduce HM concentration and improve grain yield at the HMP level. We suggest that Si foliar can be used to enhance plant growth and productivity in irrigation with HMP [63]. Moreover, Edelstein (2018) and Zhan (2018) reported that arbuscular mycorrhizal fungi (AMF) could induce resistance to HMs. Hence, the direct strategy involves using AMF in regions irrigated with sewage irrigation in the study area [64,65].

#### 3.6.4. Vehicular Emissions

Since 2008, the consumption of gasoline and diesel has increased on a yearly basis, from 2.11 million tons of gasoline to 4.949 million tons in 2016 and from 5.317 million tons of diesel in 2008 to 8.436 million tons in 2016 [12]. The increase in gasoline and diesel directly reflects the increase in car ownership. Pb and Cu were the primary factors responsible for air pollution [20,66,67,68].

Research results demonstrated that the HM concentration in roadside soil was zoned along the distance and decreased exponentially with an increase in the roadside distance [69,70,71,72,73]. For instance, MacKinnon (2011) reported that the Pb accumulation range caused by traffic activities was limited to 10 m of the expressway and 3 m of feeder road [67]. In future, we should focus on monitoring the content of HMs in arable soil within 10 m of the roadside. Moreover, green vegetation enrichment is an economical and effective technique to reduce HMP in the atmosphere [70]. Therefore, increasing the green belt or changing the planting structure of the green belt can improve the concentration of HMs in arable soil. Karmakar (2019) evaluated fifteen plants using the air pollution tolerance index, expected performance index, and metal accumulation index to determine their tolerance to air pollution, expected performance, and metal accumulation capacity [71]. Esfandiari (2020) and Mondal (2021) reported that common plant species as green belts could be accumulated to improve the HM concentration level [72,73].

Moreover, the relevance and effectiveness of remote sensing technology for the on-site identification of high-emission vehicles for inspection and maintenance plans should be considered in future [41].

## 4. Conclusions

The RI result of HMs demonstrated moderately potential ecological risk. In particular, the distribution map demonstrated that the concentration of HMs in the JZN plain is greater than that in the JD plain. The high values of As, Cu, Pb, Cd, and Hg were mainly distributed in the eastern BD. Meanwhile, the input of atmospheric deposition in the Hebei plain demonstrated significant regional and industrial characteristics. The input fluxes of atmospheric deposition, irrigation water and fertilizer are As (70.91 g/hm^2^·a), Cr (275.81 g/hm2·a), Ni (138 g/hm^2^·a), Cu (419.37 g/hm^2^·a), Pb (475.11 g/hm^2^·a), Cd (17.49 g/hm^2^·a), Hg (1.33 g/hm^2^·a), and Zn (2609.9 g/hm^2^·a). The input of atmospheric deposition plays an important role in the Hebei plain. The input of irrigation water and fertilizer also showed a contribution rate for As (24%) and Hg (29%). The result of the source of HMs in the study area demonstrated traffic, fertilizer application, farming—Cd and Zn, and coal-burning—the Hg group represents the primary source of Cu, Pb, Cd, Zn, and Hg. In particular, diversified pollution can be observed based on the contribution rate of As. In general, the three input sources of atmospheric deposition, fertilizer, and irrigation water served as sinks for HMs in the Hebei plain, except for Ni and Cr. Hence, the relevant department must formulate strategies to control the input of HMs. In future, speciation in the atmosphere is important to the department of environment to perform the detailed analysis of the pollution characteristics (vehicular traffic and industrial emission), including mineral components.

## Figures and Tables

**Table 1:** Descriptive statistical analysis of the HMs in the study area (in mg/kg).

Statistical	As	Cu	Pb	Cd	Ni	Cr	Hg	Zn	pH	orgC %	CEC cmol/kg
Mean	9.38	24.77	24.48	0.19	27.62	66.65	0.06	75.62	8.15	1.03	11.23
Median	9.22	23.70	23.50	0.16	27.60	67.20	0.05	72.10	8.27	0.99	10.50
Std. Deviation	3.21	14.11	9.08	0.35	6.60	11.23	0.04	53.76	0.47	0.40	4.12
coefficient of variation %	34.18	56.98	37.07	183.02	23.88	16.85	64.65	71.08	5.77	38.83	36.69
Skewness	0.96	11.07	8.50	11.66	−0.70	−0.27	4.40	12.55	−1.52	1.81	0.82
Kurtosis	4.87	156.19	88.87	137.08	1.29	3.65	27.34	180.81	3.06	7.98	0.66
Minimum	2.47	5.60	13.70	0.05	5.40	25.00	0.01	15.80	6.20	0.16	2.70
Maximum	29.50	228.90	125.70	4.52	43.20	112.10	0.36	879.00	9.05	3.66	27.40
Local background [29]	12.80	21.80	21.50	0.09	30.80	68.30	0.04	71.90	-	-	-
Soil risk screening values [35]	25.00	100.00	170.00	0.60	190.00	250.00	3.40	300.00	-	-	-

Ref. [29] Chinese soil element background value 1990. Ref. [35] Soil environmental quality GB15618-2018.

**Table 2:** The Igeo and RI mean values of the HMs in the study area.

Heavy Metals	As	Cu	Pb	Cd	Ni	Cr	Hg	Zn
Igeo	−0.34 ± 0.16	−0.15 ± 0.14	−0.14 ± 0.09	−0.008 ± 0.16	−0.24 ± 0.13	−0.19 ± 0.08	−0.05 ± 0.19	−0.18 ± 0.13
Er	9.52 ± 3.25	4.88 ± 2.78	5.62 ± 2.08	52.46 ± 96.02	4.35 ± 1.04	1.92 ± 0.32	133.01 ± 85.99	1.13 ± 0.80
RI	212.90 ± 142.55							

## Supplementary Materials

The following are available online at https://www.mdpi.com/article/10.3390/ijerph19042288/s1, Figure S1: The Cluster analysis combine map of heavy metals in study area by using Average Linkage (Between Groups), Table S1: Correlation analysis of heavy metals in the study area by the Pearson, Table S2: Rotated component matrix, Table S3: The mean values of Heavy metals in chemical fertilizers (mg/kg).
