# Supplementary material for: Ecological Risk, Input Flux, and Source of Heavy Metals in the Agricultural Plain of Hebei Province, China
Source: Int J Environ Res Public Health. 2022 Feb 17;19(4):2288. doi: 10.3390/ijerph19042288 (PMC8872327; doi:10.3390/ijerph19042288)
Supplement: Supplementary file 1 [file ijerph-19-02288-s001.zip › ijerph-1562493-supplementary.pdf]

Ecological Risk, Input Flux, and Source of Heavy Metals in Agricultural Plain in Hebei Province, China

TableS1 Correlation analysis of trace elements in the all area by the Pearson method

Heavy metals	As	Cu	Pb	Cd	Ni	Cr	Hg	Zn
As	1	0.550**	0.414**	0.289**	0.671**	0.513**	0.059	0.259**
Cu	0.550**	1	0.687**	0.644**	0.451**	0.388**	0.260**	0.396**
Pb	0.414**	0.687**	1	0.561**	0.334**	0.310**	0.226**	0.388**
Cd	0.289**	0.644**	0.561**	1	0.116	0.080	0.148*	0.879**
Ni	0.671**	0.451**	0.334**	0.116	1	0.869**	0.090	0.261**
Cr	0.513**	0.388**	0.310**	0.080	0.869**	1	0.224**	0.216**
Hg	0.059	0.260**	0.226**	0.148*	0.090	0.224**	1	0.135*
Zn	0.259**	0.396**	0.388**	0.879**	0.261**	0.216**	0.135*	1

**. Correlation is significant at the 0.01 level (2-tailed).

*. Correlation is significant at the 0.05 level (2-tailed).

Table S2. Rotated component matrix.[a]

Elements	Extraction	Component			
		1	2	3	4
As	0.726	0.661	0.510	0.076	-0.154
Cu	0.845	0.284	0.819	0.278	0.131
Pb	0.80	0.147	0.841	0.233	0.130
Cd	0.978	-0.031	0.470	0.869	0.037
Ni	0.943	0.950	0.183	0.080	0.001
Cr	0.897	0.920	0.089	0.061	0.198
Hg	0.969	0.070	0.138	0.055	0.971
Zn	0.982	0.165	0.127	0.968	0.048
Total		2.321	1.935	1.841	1.043
% of Variance		29.011	24.194	23.014	13.041
Cumulative %		29.011	53.205	76.219	89.260

Extraction Method: Principal Component Analysis.
Rotation Method: Varimax with Kaiser Normalization.
a. Rotation converged in 5 iterations.

TableS3 The mean values of Heavy metals in chemical fertilizers, irrigation water and atmospheric deposition（mg/kg）

type		As	Cd	Cr	Cu	Hg	Ni	Pb	Zn
Chemical fertilizers	compound fertilizer	2.59	0.67	11.51	3.90	0.041	6.05	3.63	41.78
	Urea	0.80	0.04	1.16	0.10	0.005	1.05	0.51	1.01
	ammonium hydrogen carbonate	0.74	0.020	2.90	0.40	0.005	3.26	0.020	1.95
	phosphate fertilizer	9.08	0.58	9.49	10.6	0.040	6.98	2.33	44.7
Irrigation water(mg/L)		0.0029	0.0008	0.0027	0.0029	—	0.0056	0.0102	0.0088
Atmospheric deposition		13.36	4.93	65.95	57.68	0.31	30.89	141.96	1263.64
